# Repurposing of Drugs for Antibacterial Activities on Selected ESKAPE Bacteria *Staphylococcus aureus* and *Pseudomonas aeruginosa*

**DOI:** 10.1155/2020/8885338

**Published:** 2020-09-29

**Authors:** Bridget Kamurai, Molly Mombeshora, Stanley Mukanganyama

**Affiliations:** Department of Biochemistry, University of Zimbabwe, P.O. Box MP 167 Mt. Pleasant, Harare, Zimbabwe

## Abstract

Increasing cases of multidrug-resistant pathogens have evolved into a global health crisis. ESKAPE group of bacteria are associated with antibiotic resistance, and infections caused by these pathogens result in high mortality and morbidity. However, *de novo* synthesis of antibiotics is expensive and time-consuming since the development of a new drug has to go through several clinical trials. Repurposing of old drugs for the treatment of antimicrobial resistant pathogens has been explored as an alternative strategy in the field of antimicrobial drug discovery. Ten non-antimicrobial compounds were screened for antibacterial activity on two ESKAPE organisms, *Staphylococcus aureus* and *Pseudomonas aeruginosa*. The drugs used in this study were amodiaquine an antimalarial drug, probenecid used to prevent gout, ibuprofen a painkiller, 2-amino-5-chlorobenzaxazole used as a tool for assessing hepatic cytochrome P450 activity in rodents, ellargic acid an antioxidant, quercetin an antioxidant and anti-inflammatory drug, N–N diacryloylpiperazine used to crosslink polyacrylamide gel in 2D-protein electrophoresis, epicatechin an antioxidant and antiviral drug, curcumin an anticancer drug, and quinine an antimalarial drug. Antibacterial susceptibility tests were carried out for the 10 compounds. Curcumin exhibited the most potent antimicrobial activity against both bacteria, with MICs of 50 *μ*g/ml and 100 *μ*g/ml for *P. aeruginosa* and *S. aureus*, respectively. Ellargic acid was found to have an MIC of 100 *μ*g/ml against *S. aureus.* Curcumin caused protein and nucleic acid leakage from the bacterial cell membrane in both bacterial species. When curcumin was combined with ciprofloxacin, it was found to enhance the antibacterial effects of ciprofloxacin. The combination with ciprofloxacin reduced the MIC for ciprofloxacin from 0.5 *μ*g/ml to 0.0625 *μ*g/ml on *P. aeruginosa* and 0.25 *μ*g/ml to 0.0625 *μ*g/ml on *S. aureus*. The results obtained show that curcumin has antibacterial activity against *S. aureus* and *P. aeruginosa* and may enhance the antibacterial activity of ciprofloxacin.

## 1. Introduction

A group of six organisms has been labelled as ESKAPE pathogens (*Enterococcus faecium, Staphylococcus aureus, Klebsiella pneumoniae, Acinetobacter baumannii, Pseudomonas aeruginosa*, and *Enterobacter* spp. [1]. The bacteria are common causes of life-threatening nosocomial infections amongst critically ill and immunocompromised individuals and are characterised by potential resistance mechanisms [2]. The resistant forms of the ESKAPE bacteria are associated with poor clinical outcomes compared with their susceptible counterparts. Antimicrobial resistance has developed into a serious threat to the public health in every geographic region. Antimicrobials have been crucial allies in the treatment of bacterial infections for almost 80 years [3]. However, multidrug-resistant strains of microbes have emerged resulting in some antimicrobials becoming ineffective [4]. This calls for the urgent development and discovery of new drugs. However, this is quite a long and expensive process. The new drug has to undergo a number of clinical trials before it is released into the market.

Drug repurposing is one strategy that warrants attention as a unique method for development of new antimicrobials. The strategy is based on rediscovering new applications outside the scope of the original medical indication of the drug [5]. These drugs have already been tested in humans, and their safety, efficacy, pharmacokinetic parameters, and toxicity profiles have been extensively studied. Therefore, this permits a better understanding of the overall pharmacology of the drug, potential routes of administration, and the best way to establish an appropriate dosing regimen. Repurposing reduces costs and time since the new drug is allowed to bypass some of the clinical trials. Researchers have mined existing libraries of clinical molecules in order to repurpose old drugs for new applications as antimicrobials. A number of drugs have been successfully repurposed, and an example is auranofin. Auranofin is a drug initially approved as an antirheumatic agent, which also possesses potent antibacterial activity in a clinically achievable range. Auranofin was found to possess potent antibacterial activity against *S*. *aureus* [6]. The in vitro MIC reported for this drug ranges from 0.125 mg/ml to 0.5 mg/ml. More importantly, auranofin demonstrated bactericidal activity against several multidrug-resistant strains of *S*. *aureus* within an achievable clinical drug concentration in humans. Ebselen an organoselenium compound has been widely investigated for its anti-inflammatory, antiatherosclerotic, and antioxidative properties. Ebselen also proved to possess potent antimicrobial activity against vancomycin-resistant enterococci [7].

*S*. *aureus* is a Gram-positive bacterium found in the environment as well as in normal human flora, located on the skin and mucous membranes (most often the nasal area) of most healthy individuals [8]. The bacterium has demonstrated a unique ability to quickly respond to each new antibiotic with the development of a resistance mechanism, including penicillin and methicillin, linezolid, and daptomycin [9]. The incidence of community-acquired and hospital acquired *S*. *aureus* infections has been rising with increasing emergency of drug-resistant strains called methicillin-resistant *S*. *aureus* (MRSA). MRSA has developed into a global problem, being among the most common causes of hospital acquired infections. *P*. *aeruginosa* is an opportunistic pathogen implicated in respiratory infections, urinary tract infections, gastrointestinal infections, keratitis, and bacteremia in patients with compromised host defenses [10]. *P*. *aeruginosa* has proved to cause serious therapeutic challenges for the treatment of community and hospital-acquired infections [11].

Multidrug-resistant pathogens have become a significant threat to public health and a challenge to healthcare providers due to diminishing numbers of effective antibiotics resulting in development of complicated infections that are difficult to treat. *S*. *aureus* and *P*. *aeruginosa* infections have caused serious complications. This calls for urgent action to develop new antibiotics. However, this is quite an expensive and time-consuming process. The objective of this study was to determine the effects of 10 existing, marketed, nonantibiotics for antibacterial activities against *S*. *aureus* and *P*. *aeruginosa*.

## 2. Methods and Materials

### 2.1. Reagents and Materials

All chemicals used in the study were obtained from Sigma-Alrdich (Steinheit, Germany) including ten non-antimicrobial drugs, amodiaquine, quercetin, quinine, curcumin, ellargic acid, 2-amino-5-chlorobenzaxazole, N–N diacrylolpiperazine, epicatechin, ibuprofen, and probenecid. The strains used were laboratory strains of *P. aeruginosa* (ATCC 27853) and *S. aureus* (ATCC 9144). The bacterial strains were obtained from the Department of Biological Science at the University of Botswana. Both strains are susceptible towards ciprofloxacin.

### 2.2. Antibacterial Susceptibility Testing

Antibacterial susceptibility tests were carried out according to [12]. Single colonies of bacteria were picked from an agar plate and inoculated in Luria broth media. The bacterial suspension was incubated overnight. Bacterial suspension was adjusted to an equivalent of 0.5 McFarland's standard. The concentration of cells was adjusted to 1 × 10^6^ cfu/ml by diluting with media. Antibacterial effects of the compounds were tested using the broth microdilution assay. Each compound was dissolved in DMSO. Concentrations of 0 *μ*g/ml, 12.5 *μ*g/ml, 25 *μ*g/ml, 50 *μ*g/ml, and 100 *μ*g/ml of the compounds were prepared from stock solution. Ciprofloxacin was used as the standard antibiotic. A volume of 100 *μ*l of test compound and 100 *μ*l of cells were added to wells onto a 96-well plate making a total of 200 *μ*l in each well. This was followed by 24 hour incubation. After 24 hour incubation, MTT assay was carried out to determine the cell viability. A volume of 20 *μ*l of MTT reagent was added to every well and incubated for 2 hours. A dark purple color indicated presence of viable cells. [13]. Absorbance was read at 590 nm using a Tecan Genios-Pro microplate reader (Tecan Group Ltd Mannedorf, Switzerland). Minimum inhibitory concentration (MIC) was determined, the lowest concentration that exhibited absence of viable cells seen as a yellow color of the MTT. Cell viability was calculated and expressed as a percentage.

#### 2.2.1. Checkerboard Assay and Time-Kill Assay

Bactericidal properties of the test compounds were assessed using a time-kill assay [14]. The bactericidal properties were tested by broth microdilution, performed on 96-well plates. The plates were incubated at 37ºC, and absorbance was measured at 590 nm using a Tecan Genios-Pro microplate reader after time 2 hours, 4 hours, 8 hours, 24 hours, 28 hours, and 32 hours. The checkerboard assay was performed as described by Chang et al. [14]. Varying concentrations of curcumin (25, 50, and 100 *μ*g/ml) and ciprofloxacin (0.0625, 0.125, 0.25, 0.5, and 1 *μ*g/ml) were combined to investigate the effect of the combined drugs on *P. aeruginosa* and *S. aureus*.

#### 2.2.2. Determination of the Effect of Compound Curcumin on Bacterial Nucleic Acid Leakage

Propidium iodide, a dye that is capable of binding to nucleic acids, was used to investigate the effects of the drugs on bacterial membranes as described by Moyo and Mukanganyama [15]. The dye is unable to enter viable cells. *P. aeruginosa* and *S. aureus* cells were suspended in 0.9% saline solution (OD600 = 1.5). The cell suspensions were exposed to different concentrations of the drugs, half the MIC (½ MIC), MIC, and twice the MIC (2 × MIC) in duplicate for 10 minutes. 1 ml of the bacterial suspension was centrifuged for 1 minute at 11 000 rpm. The pellet was washed with 1 ml 0.9% saline solution. A volume of 3 *μ*l of propidium iodide was added to each sample, the solution was mixed, and samples were kept in the dark for 10 minutes. Fluorescence was measured at excitation and emission wavelengths of 544 nm and 612 nm, respectively, using a *f*max microplate spectrofluorometer (Molecular Devices, Sunnyvale, USA). The controls used were untreated cells and 0.1% sodium dodecyl sulphate (SDS).

#### 2.2.3. Determination of the Effect of Curcumin on Bacterial Protein Leakage

Cells were suspended in 0.9% saline solution (OD_600_ = 1.5). Cell suspensions were exposed to drug curcumin at concentrations of 1/2 MIC, MIC, and 2 × MIC. Samples were incubated at 37ºC with shaking (120 rpm) for 120 minutes. A volume of 500 *μ*l cell suspension was centrifuged at 7000 rpm for 2 minutes. The protein content was determined using Bradford's method. Briefly, 950 *μ*l of coomassie brilliant blue G-250 was added to 50 *μ*l of the supernatant. The color was allowed to develop for 10 minutes, and absorbance was measured at 590 nm using a Tecan Genios-Pro microplate reader. The controls used were 0.1% SDS and untreated cells. Bovine serum albumin (BSA) was used as a standard to determine protein concentration.

### 2.3. Statistical Analysis

Statistical analyses were carried out using GraphPad Prism version 6. The data were expressed in the form of mean ± standard deviation of the mean. Statistically significant differences between various means of controls and the tests were analysed using the one-way ANOVA and Dunnett's multiple comparison posttest with a *p* value at *p* < 0.05 is considered significant.

## 3. Results

### 3.1. Screening for Antibacterial Activities of Non-Antimicrobial Agents

**Figure 1:**
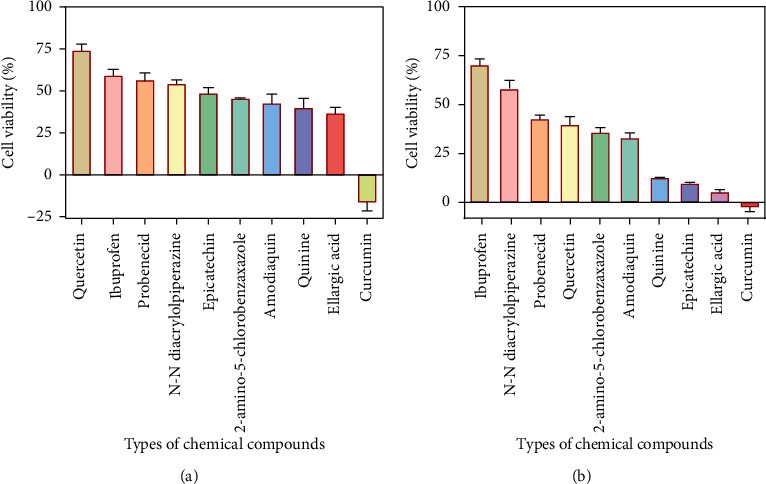
Effects of 10 non-antimicrobial drugs on *P. aeruginosa* (a) and *S. aureus* (b). Antimicrobial activity of the drugs is shown by the percentage of viable cells after exposure to drugs at 100 *μ*g/ml.

The 10 non-antimicrobial drugs under the study were tested for antibacterial activities against *P. aeruginosa* and *S. aureus*, and the percentage cell viability of each drug is shown in Figures 1(a) and 1(b), respectively.

For *P. aeruginosa* (Figure 1(a)), curcumin exhibited the most potent antibacterial activity with a percentage cell viability of −16.6%. For *S. aureus* (Figure 1(b)), curcumin showed the highest antibacterial activity with a percentage cell viability of −2.98%. A detailed percentage inhibition summary and structures for the 10 compounds is shown in Table 1.

### 3.2. Determination of MIC of Curcumin against *P. aeruginosa* on *S. aureus*

**Figure 2:**
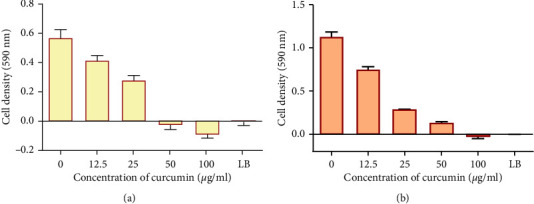
The effect of curcumin on growth of *P. aeruginosa* (a) and *S. aureus* (b). Values are expressed as mean cell density at 590 nm wavelength ± the standard deviation (*n* = 4). LB is Luria broth media and served as the negative control.

Having determined that, at a concentration of 100 *μ*g/ml, curcumin was the most potent antibacterial effects against *P. aeruginosa on S. aureus*, further tests were carried out using curcumin. The MIC of curcumin against *P. aeruginosa* and *S. aureus* cell was determined using the broth microdilution assay. Antibacterial activities of curcumin increased with increasing concentration of drug for both bacteria. MIC of curcumin was at 50 *μ*g/ml against *P. aeruginosa* ((Figure 2(a)) and 100 *μ*g/ml against *S*. *aureus* (Figure 2(b)).

Antibacterial activity of the standard drug ciprofloxacin increased with increasing concentration of drug. MICs of 0.5 *μ*g/ml and 0.25 *μ*g/ml were obtained for ciprofloxacin against *P. aeruginosa* and *S. aureus*, respectively.

### 3.3. Effects of Combining Curcumin and Ciprofloxacin on *P. aeruginosa* and *S. aureus*

**Figure 3:**
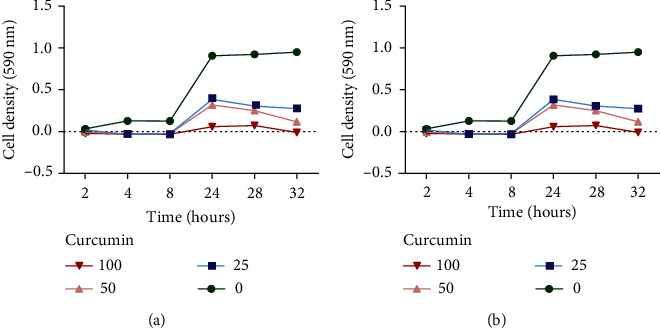
The time-kill effects of combining ciprofloxacin and curcumin against *P. aeruginosa* (a) and *S. aureus* (b).

A checkerboard assay combined with a time-kill assay was carried out to investigate the effects of combining curcumin with ciprofloxacin for different time intervals over a period of 32 hours. Curcumin was found to enhance the antibacterial effects of ciprofloxacin against *P. aeruginosa* and *S. aureus* as shown in Figures 3(a) and 3(b), respectively.

The checkerboard assay was carried out to determine the effects combining of the two compounds [16] on the MIC of the standard drug. Combining curcumin with ciprofloxacin resulted in a decrease in the MIC of ciprofloxacin against *P. aeruginosa* and *S. aureus* as shown in Table 2.

Curcumin was found to enhance the antibacterial activities of ciprofloxacin against *P. aeruginosa* and *S*. *aureus*.

### 3.4. Effects of Curcumin Nucleic Acid Leakage and Protein Leakage

**Figure 4:**
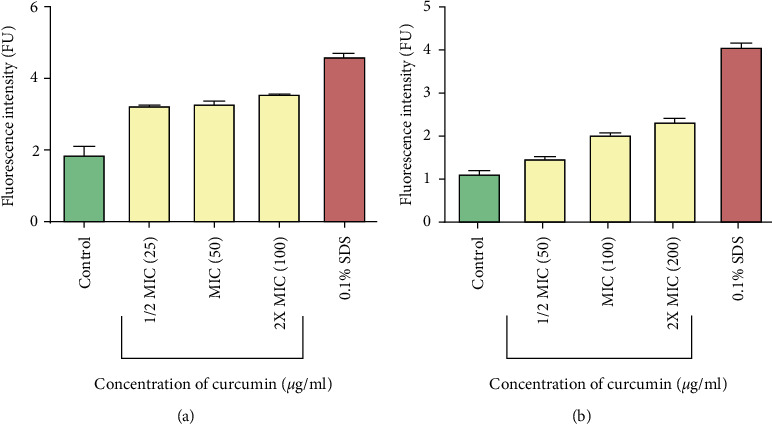
Fluorescence of propidium iodide bound to nucleic acids of *P. aeruginosa* (a) and *S. aureus* (b) cells after exposure to different concentrations of curcumin. Cells with no extract were used as the control. Values are for mean ± standard deviation (error bar) for *n* = 2.

To determine the possible mechanism of action employed by curcumin in inhibiting the growth of bacteria, its effects on nucleic acid leakage and protein leakage were determined. Cells were exposed to different concentrations of curcumin (1/2 MIC, MIC, and 2 x MIC). It was shown that in both cases of the bacteria, there was increased nucleic acid leakage upon exposure to curcumin (Figure 4). Fluorescence intensity increased with increasing drug concentration.

**Figure 5:**
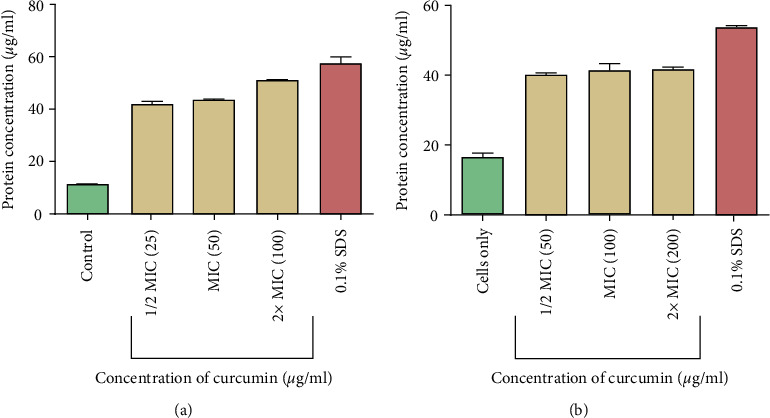
The effect of curcumin on protein leakage from *P. aeruginosa* (a) and *S. aureus* (b) cells. The test was done in triplicate.

The amount of protein leaked out of bacterial cells after exposure to curcumin was determined using the Bradford method. It was shown that there was increased protein leakage as the protein concentration was increased from 1/2 MIC to 2 x MIC (Figure 5).

## 4. Discussion

The Infectious Diseases Society of America (ISDA) formulated an acronym ESKAPE, in order to emphasize the group of pathogens that cause hospital infections and effectively “escape” the effects of antibacterial drugs [17]. These include the Gram-positive organisms vancomycin-resistant enterococci (VRE) and methicillin–resistant *S*. *aureus* (MRSA); the Gram-negative pathogens *P*. *aeruginosa*, *Acinetobacter baumanii*, and extended spectrum-*β*-lactamase producing (ESBL), or carbapenem-resistant eneterobacteriaceae (CRE). The bacteria are common causes of life-threatening nosocomial infections amongst critically ill and immunocompromised individuals and are characterised by potential resistance mechanisms [2].

The main interest of the study was to explore non-antimicrobial drugs that would have exhibited the most potent antimicrobial activity against two ESKAPE pathogens *P*. *aeruginosa* and *S*. *aureus.* It was shown that 2 compounds ellargic acid and curcumin had significant antimicrobial activity on both bacteria. Ellargic acid had an MIC of 100 *μ*g/ml against *S. aureus*. Curcumin gave an MIC of 50 *μ*g/ml and 100 *μ*g/ml on *P. aeruginosa* and *S. aureus*, respectively. Curcumin exhibited the most potent antimicrobial activity against both pathogens under study. Antimicrobial activities of curcumin observed in this study were consistent with the previously published findings [18, 19]. Curcumin inhibited the growth of *S. aureus* and *P. aeruginosa* cells but did not kill the bacteria thus exhibiting bacteriostatic properties [20]. Ellargic acid inhibited the growth of *S. aureus*, and ellargic acid is known to contain polyphenols that may be responsible for the observed antibacterial activity [21]. A number of studies have shown that plants that contain polyphenols are a good source of antimicrobial agents against bacteria, viruses, and protozoa [22].

Combined therapy has been reported of being a useful alternative in the field of antimicrobial drug development [23]. Curcumin was found to enhance the antibacterial activity of ciprofloxacin on both bacteria. There was a reduction of the MIC of ciprofloxacin when tested against *P. aeruginosa* from 0. 5 *μ*g/ml to 0.0625 *μ*g/ml. Exposure of *S. aureus* to a combination of curcumin and ciprofloxacin resulted in reduction of the MIC of ciprofloxacin from 0.25 *μ*g/ml to 0.0625 *μ*g/ml. The results suggested that a combination of curcumin and ciprofloxacin enhanced its antibacterial effects on *P. aeruginosa* and *S. aureus.* These results correlated with previously published studies, where curcumin was found to enhance the activities of antifungal [24], anticancer [25], and antibacterial [22] agents. It has been documented that co-treatment of cancer cells with curcumin and cisplatin results in a substantial increase in cancer cell death as compared with cisplatin treatment alone [26].

Few studies have demonstrated the mechanism of antibacterial activity of curcumin which seems to differ depending on the strain being studied [20, 27]. Studies have demonstrated that curcumin inhibits cytokinesis and bacterial proliferation on some bacteria, e.g., *Bacillus subtilis* [28]. Curcumin is suspected of binding to Ftsz proteins resulting in the inhibition of Ftsz protofilaments which in turn suppresses the formation of the z-ring leading to the inhibition of cytokinesis and bacterial proliferation [29]. Curcumin was also reported of having the ability to increase sensitivity of bacteria towards *β*-lactam antibiotics. In the case of MRSA, curcumin was suggested to inhibit the mecA gene transcription causing reduced expression of PBP2*α* proteins. As a result MRSA can be sensitised towards the antibacterial action of *β*-lactam antibiotics, e.g., penicillin and methicillin [30].

In this study, to investigate the mechanism of action employed by curcumin to inhibit growth of *P. aeruginosa* and *S. aureus*, protein leakage and nucleic acid leakage assays were carried out. This was done in order to determine the effects of curcumin on the integrity of bacterial membrane. Gram-negative bacteria have outer and inner membranes, though the thickness of the membrane is less than that of Gram-positive bacteria. The outer membrane is lipopolysaccharides (LPSs) in nature. Gram-negative bacteria are resistant towards hydrophobic antibiotics and toxic drugs. The inner membrane or cell wall of Gram-negative bacteria is protected from antibiotics by the outer membrane [31]. Treatment of *P. aeruginosa* and *S. aureus* cells with curcumin resulted in the leakage of proteins and nucleic acids, and this was an indication of membrane damage. The extent of damage can be evaluated from the amount of the released cellular components [32]. Nucleic acid leakage was determined by adding propidium iodide dye to cells after exposure to curcumin. PI is membrane impermeable and is generally excluded from viable cells. PI is commonly used for identifying dead cells in a population and as a counterstain in multicolor fluorescent techniques [20]. PI binds to nucleic acids and in turn fluoresces. The fluorescence intensity is directly proportional to the amount of nucleic acids that would have leaked. Increase in fluorescence in curcumin treated cells clearly indicated membrane disruption. Leakage of cytoplasmic proteins and nucleic acids suggests that curcumin disrupted the bacterial cell membrane. Bacterial membranes are composed of lipid, protein, and lipoproteins. The cytoplasmic membrane acts as a diffusion barrier for water, ions, nutrients, and transport systems. Disorganisation of the membrane permeability results in protein and nucleic acid leakage. Targeting the membrane is a crucial strategy of an antimicrobial drug development [33]. Therapeutic molecules having potential to damage membrane may thus be used as an antimicrobial drug even for bacteria causing persistent infection [30].

Other studies have reported that curcumin has poor bioavailability and selectivity [34]. To improve the bioavailability of curcumin, to provide longer circulation, and to increase the cellular permeability and resistance to metabolic processes, adjuvants have to be developed and introduced. An example is piperine that interferes with glucuronidation of curcumin in the liver [35]. Once in the blood stream, curcumin and ciprofloxacin combination may overcome the resistance to ciprofloxacin in MRSA or *P*. *aeruginosa* drug-resistant strains.

## 5. Conclusion

The antimicrobial activities of the 10 non-antimicrobial compounds against *S. aureus* and *P. aeruginosa* were determined. Curcumin exhibited the most potent antimicrobial activity against *S. aureus* and *P. aeruginosa.* Curcumin may act by targeting the bacterial cell membrane causing leakage of proteins and nucleic acids. Curcumin was also shown to enhance the antibacterial activities of ciprofloxacin. Further studies are required to determine the effects of combining curcumin with other antibiotics. Results of this study suggest curcumin to be an attractive candidate for repurposing as an antibacterial drug.

## Figures and Tables

**Table 1:** Structures of tested non-antimicrobial drugs screened for antibacterial effects on *S*. *aureus* and *P*. *aeruginosa*, MIC, and % cell viability.

Drug	Organism	MIC (*μ*g/ml)	% cell viability
Probenecid	*S*. *aureus*	—	42.8
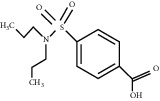	*P*. *aeruginosa*	—	56.9
Amodiaquine	*S. aureus*	—	33
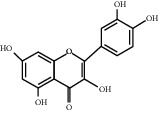	*P*. *aeruginosa*	—	43.2
2-Amino-5-chlorobenzaxazole	*S. aureus*	—	34.7
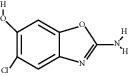	*P*. *aeruginosa*	—	46
Quercetin	*S. aureus*	—	40
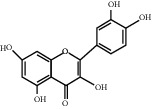	*P*. *aeruginosa*	—	65.9
Ibuprofen	*S. aureus*	—	70.6
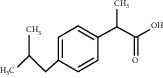	*P*. *aeruginosa*	—	59.3
Epicatechin			
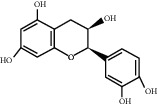	*S. aureus*	—	7.1
	*P*. *aeruginosa*	—	49
Ellargic acid	*S. aureus*	100	4.9
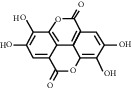	*P*. *aeruginosa*	—	36.8
Curcumin	*S. aureus*	100	
	−2.98		
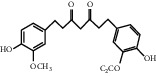	*P*. *aeruginosa*	50	−16.6
N–N Diacryloylpiperazine	*S. aureus*	—	58
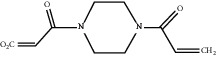	*P*. *aeruginosa*	—	54
Quinine	*S. aureus*	—	13.1
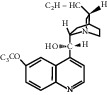	*P*. *aeruginosa*	—	40.3
Ciprofloxacin	*S. aureus*	0.25	−0.8
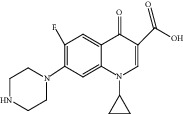	*P*. *aeruginosa*	0.5	−1.1

“–” indicates no MIC.

**Table 2:** The effects of combining ciprofloxacin and curcumin on the MIC of ciprofloxacin against *P. aeruginosa* and *S. aureus*.

Bacteria	MIC of ciprofloxacin only (*μ*g/ml)	MIC of ciprofloxacin combined with curcumin (*μ*g/ml)
*P. aeruginosa*	0.5	0.0625
*S. aureus*	0.25	0.0625

## Data Availability

The datasets used and/or analysed during the current study are available from the corresponding author on reasonable request.
